# Serum levels of soluble TNF-α receptors but not BDNF are associated with apathy symptoms in mild Alzheimer's disease and amnestic mild cognitive impairment

**DOI:** 10.1590/S1980-57642013DN70300011

**Published:** 2013

**Authors:** Henrique Cerqueira Guimarães, Paulo Caramelli, Patricia Paes Araujo Fialho, Elisa de Paula França, Marcelo Pelizzaro Dias Afonso, Antonio Lucio Teixeira

**Affiliations:** 1 Behavioral and Cognitive Neurology Research Group, Department of Internal Medicine, Faculty of Medicine of the Federal University of Minas Gerais, Belo Horizonte (MG), Brazil.; 2 Translational Psychoneuroimmunology Group, Department of Internal Medicine, Faculty of Medicine of the Federal University of Minas Gerais, Belo Horizonte (MG), Brazil.

**Keywords:** apathy, dementia, Alzheimer's disease, mild cognitive impairment, TNF-α, sTNFR1, sTNFR2, BDNF

## Abstract

Apathy is intimately associated with dementia. Unfortunately, its pathophysiology remains poorly understood. The motivational impairment that characterizes this disorder might share the same inflammatory mechanisms, as suggested by the sickness behavior theory.

**OBJECTIVE:**

The primary aim of this study was to investigate the association between apathy symptoms and serum levels of tumor necrosis factor alpha (TNF-α) and its soluble receptors. Brain-derived neurotrophic factor (BDNF) levels were also analyzed since these have been associated with depression, a condition which shares abulic features with apathy.

**METHODS:**

The sample consisted of 27 subjects with mild Alzheimer's disease or amnestic mild cognitive impairment, who were submitted to specific apathy evaluation using the Apathy Scale (AS) and provided blood samples for biomarker analysis. Participants were categorized into two groups according to median AS scores (17 points).

**RESULTS:**

Subjects with higher apathy symptoms (n=13) displayed higher levels of TNF-α soluble receptors (type 1: p=0.03; type 2: p=0.04). No other difference was found between groups.

**CONCLUSION:**

These findings point to the involvement of inflammatory mediators in the genesis of apathy symptoms, as suggested by the sickness behavior theory.

## Abstract (pt)

Apatia está intimamente associada à demência. Lamentavelmente, sua fisiopatologia ainda é pouco compreendida. O comprometimento motivacional que caracteriza este transtorno poderia compartilhar mecanismos inflamatórios como sugere a teoria do comportamento associado à doença.

**OBJETIVO:**

O principal objetivo deste estudo foi investigar a associação entre apatia e os níveis séricos do fator de necrose tumoral alfa (TNF-α) e de seus receptores solúveis. Os níveis de fator neurotrófico derivado do cérebro também foram analisados já que estes foram associados à depressão, que compartilha aspectos abúlicos com a apatia.

**MÉTODOS:**

A amostra consistiu de 27 indivíduos com doença de Alzheimer leve ou com comprometimento cognitivo leve amnéstico, que foram submetidos à avaliação de apatia pela Escala de Apatia (EA), e proveram amostra de sangue para análise de biomarcadores. De acordo com a mediana de escores na EA (17 pontos), a amostra foi divida em dois grupos.

**RESULTADOS:**

O grupo com mais sintomas de apatia apresentou maiores níveis séricos de receptores solúveis de TNF-α (tipo 1: p=0,03 ; tipo 2: p=0,04). Nenhuma outra diferença foi encontrada entre os grupos.

**CONCLUSÃO:**

Estes achados sugerem o envolvimento de mediadores inflamatórios na gênese de sintomas de apatia, assim como sugere a teoria do comportamento associado à doença.

## INTRODUCTION

Apathy is a pervasive feature in several neu-rent understanding regarding this behavioral ropsychiatric disorders.^1^ Most of our cur-syndrome was built upon research on neurodegenerative conditions.^2^ Generally speaking, apathy has been shown to be the most prevalent behavioral disorder in dementia.^3^ Once identified, apathetic symptoms follow a prolonged and mostly definitive course throughout cognitive decline,^4^ leading to a sharp increase in apathy prevalence and severity as dementia reaches its moderate to advanced stages.^3^

There is fairly good agreement in the literature that apathy should be considered an independent syndrome in dementia, with specific clinical implications and probable worse outcomes.^5,6^ Another emerging consensus is that apathy can be recognized in a significant proportion of mild cognitive impairment (MCI) subjects, especially those regarded as amnestic.^7,8^ Longitudinal studies have suggested a substantially increased risk of dementia conversion in these MCI apathetic subjects.^9^ Unfortunately, scant therapeutic options are available to improve this devastating disorder, a fact which, at least partially, can be ascribed to a poor understanding of the neurobiological underpinnings of apathy.

Marin^10^ defined apathy as "lack of motivation, relative to the patient's previous level of functioning or the standards of his or her age and culture, not attributable to intellectual impairment, emotional distress or diminished level of consciousness". The motivational feature has remained the core diagnostic criteria in a consensus proposition to identify apathy in Alzheimer disease (AD) and other neuropsychiatric disorders.^3^ Lack of motivation is also the core feature of sickness behavior. This is a coordinated set of behavioral adaptations that also includes a neurovegetative dimension (fatigue, loss of appetite and sleep disorders), and a psychological dimension (depressed mood, anxiety and cognitive dysfunction), that are supposed to reorganize the organism's priorities to cope with infectious pathogens.^11^ TNF-α, a proinflammatory cytokine, plays a pivotal role in triggering sickness behavior.^12^ The levels of this cytokine have also been shown to be increased in a sample of MCI and AD patients.^13^ Since inflammatory mechanisms play a putative role in the pathophysiology of several types of neurodegeneration,^14^ and apathy is a pervasive feature in dementia, it is reasonable to investigate whether these two phenomena are associated.

The primary aim with this study was to investigate whether blood TNF-α, and its soluble receptors, are associated with apathy symptoms. Soluble forms of TNF-α receptors represent reliable markers of this cytokine activity, binding to and protecting TNF-α from proteolytic degradation, therefore, extending its effects systemically.^15^ We also investigated the possible association between BDNF and apathy, since there has been a previous report of reduced levels of this neurotrophic factor in subjects with late-life depression,^16^ a condition that shares abulic features with apathy. To accomplish this objective we evaluated only subjects with cognitive impairment in its very early stages, namely mild AD and amnestic MCI (aMCI), since inflammatory status has been related to frailty,^17,1^, an almost inexorable outcome in moderate and advanced stages of late-onset dementia.

## METHODS

**Participants and procedures.** This study was a retrospective analysis of the available clinical and laboratory data for a small subset of participants from the Pietà Study, a community-based survey of successful aging, carried out in Caeté, Southeast Brazil, in the summer of 2008. Detailed methodology has been described previously.^19^ Briefly, the study invited all of the city's inhabitants aged 75 years or older to participate, and those who agreed gave written informed consent. The study was approved by the University's research ethics committee. A total of 639 subjects were submitted to a thorough functional, clinical, psychiatric and neurological evaluation, including the Functional Activities Questionnaire (FAQ), the motor section of the Unified Parkinson's disease rating scale (UPDRSm), the M.I.N.I. structured psychiatric interview, Geriatric Depression Scale (GDS), and a Brief Cognitive Screening Battery (BCSB), consisting of the Mini-Mental State Examination (MMSE), animal category semantic fluency test, and picture drawings memory test (PDMT). Individuals suspected of having cognitive impairment, together with a subset of putative cognitively healthy control subjects, were further submitted to a comprehensive neuropsychological evaluation with the following instruments: Rey Auditory Verbal Learning Test, naming and praxis tests from the CERAD (Consortium To Establish A Registry For Alzheimer's Disease) protocol, phonemic verbal fluency tasks (FAS), Frontal Assessment Battery, and the Dementia Rating Scale. A subset of 358 subjects provided blood samples for routine and inflammatory biomarker analyses.

Among the participants submitted to the above evaluation, two groups of subjects were identified and invited for further clinical assessment pertaining to apathy and behavioral evaluation. The first group of participants consisted of 28 subjects fulfilling NINCDSADRDA diagnostic criteria for probable Alzheimer disease^20^ in the mild stage of dementia. AD staging was accomplished through the Functional Assessment Staging in Alzheimer Disease (FAST).^21^ Only those categorized as FAST 4 or below were included. None of the AD subject were taking cholinesterase inhibitors at the time of evaluations. Vascular dementia was ruled out according to NINDS-AIREN criteria.^22^ The second group consisted of 26 subjects with aMCI, diagnosed according to Petersen's criteria.^23^ Amnestic impairment was established by comparing performances on the Rey Auditory Verbal Learning Test (RAVLT) by cognitively healthy subjects from the same population. Subjects with a major depressive episode, diagnosed according to the Diagnostic and Statistical Manual of Mental Disorders fourth edition, were excluded.^24^ Of the 52 subjects with either mild AD or amnestic MCI who were submitted to apathy and behavioral evaluation, 27 underwent blood analysis, giving the final study sample reported.

**Apathy and behavioral assessment.** Since there were no published consensual diagnostic criteria for apathy at the time of data collection, this disorder was evaluated dimensionally through the Apathy Scale (AS),^25^ which was administered to caregivers or to a very close member of the household. The AS has 14 questions with a score ranging from zero to 42 points, where higher scores represent more severe apathetic symptoms. Despite a lack of consensus regarding this issue,^3^ previous studies have suggested a cut-off of 14 points in order to identify clinically relevant apathy.^25,26^ Subjects were also submitted to the Neuropsychiatric Inventory (NPI).^27^

**Laboratory assessment.** Blood was drawn during fasting and in the early morning for all the participants. Serum levels of BDNF, TNF-α, sTNF-R1, and sTNF-R2 were measured according to the procedures supplied by the manufacturer, and using ELISA kits for TNF-α (Quantikine, R&D Systems, Minneapolis, MN, USA), soluble TNF-α receptors and BDNF (DuoSet, R&D Systems, Minneapolis, MN, USA). All samples were assayed in duplicate, yielding concentrations expressed as pg/ml. The detection limits were 0.1 pg/ml for TNF-α, 10 pg/ml for both soluble receptors, and 5 pg/ml for BDNF.

**Statistical analysis.** An exploratory analysis with the D'Agostino-Pearson test showed that most of the data had non-normal distributions. The 27 eligible subjects were split into two groups according to median AS scores (17 points), forming one group with higher (AS >17) and another with lower apathy symptoms (AS ≤ 17). Differences between groups were investigated using the Mann-Whitney test for continuous variables and Chi-square test for categorical data. Correlation analysis was performed using Spearman's *rho* coefficient. Statistical significance was set as a p-value ≤0.05.

## RESULTS

The target sample consisted of 27 subjects (15 women, 12 men), with a mean age of 81.5±4.6 years, and 3.2±2.7mean years of formal education. According to cognitive status, 14 subjects presented mild AD and 13 had aMCI. The median score on the AS was 17 points, providing two groups with different intensity of apathy symptoms.

**Figure 1:**
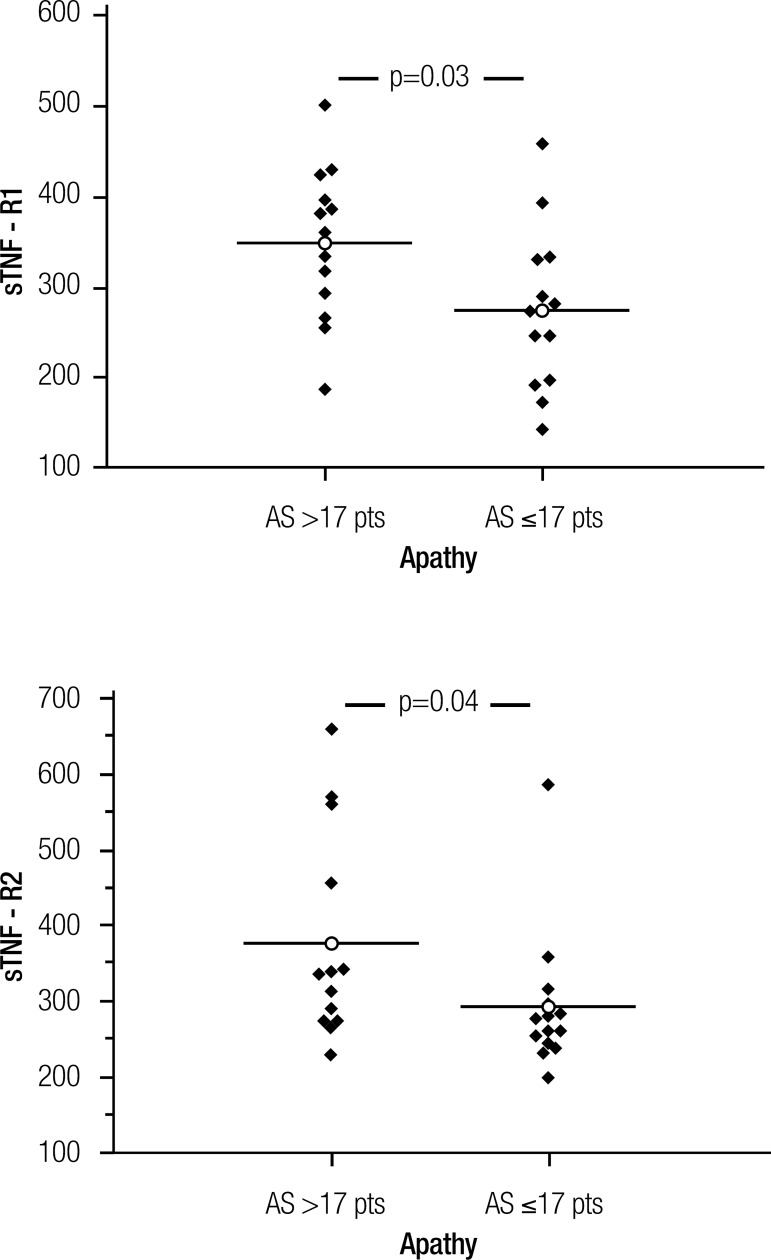
Comparison of sTNF-R1 and sTNF-R2 levels (pg/ml) between higher and lower apathy symptoms groups. AS: Apathy Scale.

Table 1 displays the comparison between the groups, regarding demographics, anthropometrics, cognitive status; cognitive, motor, functional and behavioral measures, and, lastly, BDNF and inflammatory biomarker levels. As expected, apathy measures differed between the groups. The higher apathy symptoms group had higher levels of both types of TNF-α soluble receptors (sTNF-R1: p=0.03; sTNF-R2: p=0.04). These results are illustrated in Figure 1. Soluble TNF-α receptors levels also correlated with AS scores (sTNF-R1: rho=0.49; p=0.01; sTNF-R2: rho=0.40; p=0.037), considering the whole sample. BDNF and TNF-α levels did not differ significantly between groups. No other variables reached statistical significance, except for age and NPI-total scores which displayed a tendency towards significance. Nevertheless, NPI-total scores did not correlate with levels of any of the TNF-α soluble receptor types (sTNF–R1: rho=0.05, p=0.82; sTNF-R2: rho=0.22, p=0.29). Additionally, although there was a marked difference between groups regarding proportions of gender and AD subjects, levels of TNF-α soluble receptors were not different in pairwise comparison (Mann-Whitney test), either between genders (sTNF-R1: p=0.09; sTNF-R2: p=0.3) or between different cognitive status groups (sTNF-R1: p=0.7; sTNF-R2: p=0.29), suggesting that these variables were not driving the results.

**Table 1:** Comparison of higher vs lower apathy symptoms groups, according to demographics.

	Apathy scale							p - value
	**Lower apathy symptoms (n=14)**				**Higher apathy symptoms (n=13)**			
	**%**	**mean**	**SD**		**%**	**mean**	**SD**	
Age, years	--	82.9	3.9		--	80.5	4.6	0.08
Gender (male, %)	28.6	--	--		61,5	--	--	n.s.*
Education, years	--	3.6	3.8		--	3.3	2	n.s.
Weight, Kg	--	62.9	15.9		--	67.6	13.9	n.s.
Body mass index	--	26.6	5.1		--	28.1	4.5	n.s.
Alzheimer's disease (%)	35.7	--	--		61.5	--	--	n.s.*
Cognitive and functional measures	--							
Functional Assessment Questionnaire	--	3.5	5		--	6.1	6.2	n.s.
Mini-Mental State Examination	--	20.3	4.4		--	20.4	4.8	n.s.
Dementia Rating Scale	--	100.3	19.8		--	105.1	11.7	n.s.
Frontal Assessment Battery	--	6.2	2.7		--	6.7	2.3	n.s.
Behavioral evaluation								
Apathy Scale	--	10.4	4.5		--	26.1	3.9	<0.0001
NPI - apathy	--	0	0		--	2.75	3.3	0.003
NPI - total	--	7.3	14.8		--	10.3	9.8	0.06
Geriatric Depression Scale	--	4	2.1		--	4	3.4	n.s.
Motor exam								
UPDRSm	--	6,5	6		--	9	8.3	n.s.
Biomarkers								
BDNF (pg/ml)	--	1418.3	353.9		--	1668.9	398.6	0.10
TNF-a (pg/ml)	--	227.9	546.8		--	260.5	814.8	n.s.
sTNF-R type1(pg/ml)	--	273.9	86.3		--	360.5	111.9	0.03
sTNF-R type2 (pg/ml)	--	291.6	93.8		--	377.2	138.6	0.04

NPI: Neuropsychiatric inventory; UPDRS: Unified Parkinson's disease rating scale - motor section; BDNF: brain-derived neurotrophic factor ; TNF: Tumor necrosis factor; sTNF -R: TNF soluble receptors; n.s.: non-significant ;

* Chi-square test.

## DISCUSSION

In this retrospective investigation of a small subset of subjects from the Pietà study involving participants who had provided a blood sample for inflammatory biomarker analysis and were also submitted to apathy evaluation, we found elevated levels of both soluble TNF-α receptors in subjects with greater apathy symptoms. The negative results found for TNF-α levels are unsurprising. This cytokine has a very short half-life and remains below the assay threshold in a significant proportion of subjects. As stated earlier, soluble receptors constitute more reliable markers of blood TNF-α signaling. To the best of our knowledge, this is the first study that has used a specific apathy scale to investigate association of the condition with TNF-α and its soluble receptors. A previous report found an association between GDS items, assumed to mirror apathy, and higher TNF-α levels in male patients with AD.^28^ The findings herein reported are in accordance with the sickness behavior motivational impairment theory, and might constitute a reasonable explanation for apathy ubiquity and its worsening in progressive neurodegenerative disorders. Surprisingly, very few studies have explored this association. A previous report found no relationship between a GDS surrogate apathy measure and C-reactive protein (CRP) levels.^29^ However, this acute phase reaction protein may not be the most suitable inflammatory biomarker to investigate this issue, since apathy and cognitive impairment are supposed to result from a chronic phenomenon. In line with the theoretical framework underlying this report, Ferretti et al reported, for a cohort of schizophrenic patients, a significant improvement in negative symptoms, including apathy, by treating them with minocycline,^30^ a drug with putative anti-inflammatory properties.^31^

None of the other investigated variables were found to be associated with AS scores. The two groups displayed similar depression scores, and also had similar mean performances on motor, global cognitive and executive function measures. The apparent difference regarding gender and dementia proportions between groups did not reach statistical significance, and these features were not associated with soluble TNF-α receptors. Although there was a clinically significant difference between higher and lower apathy symptoms groups regarding mean performance on the FAQ, corroborating results of a previous report,^32^ these findings did not reach statistical significance. It is important to note that BDNF levels were higher in the more apathetic group, in contrast to previous findings from a late-life depression cohort.^16^ This finding adds to a large body of evidence regarding clinical and neuroimaging data that dissociates apathy from depression.^2^

The small sample size can be considered a serious limitation of our study. Additionally, this is an emerging research field, precluding a robust hypothesis that might explain the neuroimmunology of these findings. Another important point is that the concept of MCI has proven to be, in recent years, a highly heterogeneous condition.^33^ Thus, it is reasonable to question the validity of pooling clinically defined AD and MCI groups together. However, as stated previously, inflammatory mechanisms seem to play a fundamental role in several neurodegenerative diseases, independently of their etiology, constituting a pervasive feature of dementia, akin to apathy. Lastly, the findings herein reported are preliminary and should be replicated in larger samples.

## References

[1] van Reekun R, Stuss DT, Ostrander L (2005). Apathy: why care?. J Neuropsychiatry Clin Neursoci.

[2] Guimaraes HC, Levy R, Teixeira AL, Beato RG, Caramelli P (2008). Neurobiology of apathy in Alzheimer's disease. Arq Neuropsiquiatr.

[3] Robert P, Onyike CU, Leentjens AF (2009). Proposed diagnostic criteria for apathy in Alzheimer's disease
and other neuropsychiatric disorders. Eur Psychiatry.

[4] Craig D, Mirakhur A, Hart DJ, McIlroy SP, Passmore AP (2005). A cross-sectional study of neuropsychiatric symptoms in 435
patients with Alzheimer's disease. Am J Geriatr Psychiatry.

[5] Starkstein SE, Petraca G, Chemerinski E, Kremer J (2001). Syndromic validity of apathy in Alzheimer's
disease. Am J Psychiatry.

[6] Starkstein SE, Jorge R, Mizrahi R (2006). A prospective longitudinal study of apathy in Alzheimer's
disease. J Neurol Neurosurg Psychiatry.

[7] Robert PH, Berr C, Volteau M (2006). Neuropsychological performance in mild cognitive impairment with
and without apathy. Dement Geriatr Cogn Disord.

[8] Geda YE, Roberts RO, Knopman DS (2008). Prevalence of neuropsychiatric symptoms in mild cognitive
impairment and normal cognitive aging: a population-based
study. Arch Gen Psychiatr.

[9] Robert PH, Berr C, Volteau M (2006). Apathy in patients with mild cognitive impairment and the risk of
developing dementia of Alzheimer's disease: a one-year follow-up
study. Clin Neurol Neurosurg.

[10] Marin RS (1991). Apathy: a neuropsychiatric syndrome. J Neuropsychiatry Clin Neurosci.

[11] Dantzer R (2001). Cytokine-induced sickness behavior: mechanisms and
implications. Ann N Y Acad Sci.

[12] Palin K, Bluthe RM, McCusker RH (2009). The type 1 TNF receptor and its associated adapter, FAN, are
required to TNF-α induced sickness behavior. Psychopharmcaology.

[13] Bermejo P, Martín-Aragón S, Benedí J (2008). Differences in peripheral inflammatory markers between mild
cognitive impairment and Alzheimer's disease. Immunol Lett.

[14] Amor S, Puentes F, Baker D, van der Valk P (2010). Inflammation in neurodegenerative diseases. Immunology.

[15] Wajant H, Pfizenmaier K, Scheurich P (2003). Tumor necrosis factor signaling. Cell Death Differ.

[16] Diniz BS, Teixeira AL, Talib LL, Mendonça VA, Gattaz WF, Forlenza OV (2010). Serum brain-derived neurotrophic factor level is reduced in
antidepressant-free patients with late-life depression. World J Biol Psychiatry.

[17] Hubbard RE, O'Mahony MS, Sawa GM, Calver BL, Woodhouse KW (2009). Inflammation and frailty measures in older people. J Cell Mol Med.

[18] Coelho FM, Narciso FM, Oliveira DM (2010). sTNFR-1 is an early inflammatory marker in community versus
institutionalized elderly women. Inflamm Res.

[19] Caramelli P, Barbosa MT, Sakurai E (2011). The Pietà Study: epidemiological investigation in
successful brain aging in Caeté(MG), Brazil. Methods and baseline cohort characteristics. Arq Neuropsiquatr.

[20] McKhann G, Drachman D, Folstein M, Katzman R, Price D, Stadlan EM (1984). Clinical Diagnosis of Alzheimer's disease: report of the
NINCDSADRDA work group under the auspices of department of health and human
services task force on Alzheimer's disease. Neurology.

[21]  Reisberg B (1988). Functional assessment staging (FAST). Psychopharmacol Bull.

[22] Roman GC, Tatemichi TK, Erkinjuntti T (1993). Vascular dementia: diagnostic criteria for research studies.
Report of the NINDS-AIREN International Workshop. Neurology.

[23] Petersen RC (2004). Mild cognitive impairment as a diagnostic entity. J Intern Med.

[24] American Psychiatric Association (1994). Diagnostic and Statistical Manual of Mental Disorders (DSM).

[25] Starkstein SE, Mayberg HS, Preziosi TJ, Andrezejewski P, Leiguarda R, Robinson RG (1992). Reliability, validity and clinical correlate of apathy in
Parkinsons's disease. J Neuropsychiatry Cli Neurosci.

[26] Funkiewiez A, Bertoux M, de Souza LC, Levy R, Dubois B (2012). The SEA (Social Cognition and Emotional Assessment): A clinical
neuropsychological tool for early diagnosis of frontal variant
frontotemporal lobar degeneration. Neuropsychology.

[27] Cummings JL, Mega M, Gray K, Rosenberg-Thompson S, Carusi DA, Gornbein J (1994). The Neuropsychiatric Inventory: comprehensive as sessments of
psychopathology in dementia. Neurology.

[28] Hall JR, Vo HT, Johnson LA, Winter S, Barber RC, O'Bryant SE (2011). Biomarkers and depressive symptoms in a sample of cognitively
intact and Alzheimer's disease elderly males. Neuroscience Med.

[29] Maas DW, van der Mast RC, de Craen AJ (2009). Increased C-reactive protein is not associated with apathy: the
Leiden 85-Plus Study. Int J Geriatr Psychiatry.

[30] Chaudhry IB, Hallak J, Husain N (2012). Minocycline benefits negative symptoms in early schizophrenia: a
randomised double-blind placebo-controlled clinical trial in patients on
standard treatment. J Psychopharmacol.

[31] Ferretti MT, Allard S, Partridge V, Ducatenzeiler A, Cuello AC (2012). Minocycline corrects early, pre-plaque neuroinflammation and
inhibits BACE-1 in a transgenic model of Alzheimer's disease-like amyloid
pathology. J Neuroinflammation.

[32] Boyle PA, Malloy PF, Salloway S, Cahn-Weiner DA, Cohen R, Cummings JL (2003). Executive dysfunction and apathy predict functional impairment in
Alzheimer disease. Am J Geriatr Psychiatry..

[33] Petersen RC, Aisen P, Boeve BF (2013). Criteria for mild cognitive impairment due to alzheimer's disease
in the community. Ann Neurol.

